# All-inside arthroscopic modified Broström-Gould procedure for chronic lateral ankle instability with and without anterior talofibular ligament remnant repair produced similar functional results

**DOI:** 10.1007/s00167-020-06361-2

**Published:** 2020-11-18

**Authors:** Shi-Ming Feng, Nicola Maffulli, Chao Ma, Francesco Oliva

**Affiliations:** 1 Orthopaedic Department, Sports Medicine Department, Xuzhou Central Hospital, No. 199, the Jiefang South Road, Xuzhou, 221009 Jiangsu People’s Republic of China; 2 Orthopaedic Department, Xuzhou Clinical College of Xuzhou Medical University, Xuzhou, 221009 Jiangsu People’s Republic of China; 3 Department of Musculoskeletal Disorders, Faculty of Medicine and Surgery, University of Salerno, Salerno, Italy; 4 Guy Hilton Research Centre, School of Pharmacy and Bioengineering, Keele University, Stoke-on-Trent, ST4 7QB Staffordshire UK; 5 Centre for Sports and Exercise Medicine, Barts and The London School of Medicine and Dentistry, Mile End Hospital, 275 Bancroft Road, London, E1 4DG UK

**Keywords:** Anterior talofibular ligament, Chronic lateral ankle instability, Broström-Gould procedure, Remnant repair

## Abstract

**Purpose:**

The Broström-Gould procedure, with the repair of the anterior talofibular ligament (ATFL) combined with the transfer of the extensor retinaculum, is considered the gold standard procedure for the management of chronic lateral ankle instability (CLAI). Lateral ligament reconstruction is considered if the ATFL remnant quality is poor or the ATFL has been damaged beyond the ability to suture it. It remains unclear whether not repairing the ATFL remnant produces comparable functional outcomes to the classical Broström-Gould procedure.

**Methods:**

This retrospective cohort study included 84 patients with CLAI undergoing either repair or non-repair of the ATFL remnant using an all-inside arthroscopic Broström-Gould procedure from 2015 to 2018. The Visual Analogue Scale (VAS) scores, American Orthopedic Foot and Ankle Society (AOFAS) scores, Karlsson Ankle Functional Score (KAFS), Anterior Talar Translation (ATT), Active Joint Position Sense (AJPS), and the rate of return to sports were compared in both groups.

**Results:**

All the functional scores (VAS, AOFAS, KAFS, ATT, AJPS) significantly improved in both groups at 1 and 2 years after surgery. At all the follow-up time points, the VAS, AOFAS, KAFS, ATT, AJPS, and the rate of return to sport scores were comparable between the repair and non-repair group.

**Conclusion:**

There are no statistically significant differences in postoperative outcomes between ATFL remnant repair and non-repair for the management of CLAI using the all-inside arthroscopic Broström-Gould procedure. From the clinical viewpoint, the present study shows that the potential differences in clinical outcomes between ATFL remnant repair and non-repair are likely not relevant when performing an all-inside arthroscopic Broström-Gould procedure for CLAI.

**Level of evidence:**

III.

## Introduction

Inversion injury is the most frequent ankle injury [5], with the anterior talofibular ligament (ATFL) [7] especially at risk. After an inversion injury, most patients obtain satisfactory outcomes with conservative treatment and functional rehabilitation [6, 17]. Nevertheless, surgery should be considered for patients with symptomatic ankle instability and repeated ankle sprains despite appropriate conservative management for 3–6 months [20].

The Broström-Gould procedure, with the repair of the ATFL combined with the transfer of the extensor retinaculum, is considered the gold standard procedure for the management of chronic lateral ankle instability (CLAI) [22, 25]. In this procedure, following repair of the ATFL, the extensor retinaculum is used to reinforce the lateral ankle ligament complex [18]. In some patients, the quality of the ATFL remnant is poor or the ATFL has been damaged beyond the ability to suture it, and the capsule and extensor retinaculum are the only tissues available to be repaired [13]. There could be differences in functional outcomes between repairing or not repairing the ATFL remnant using the Broström-Gould procedure. It remains unclear whether not repairing the ATFL remnant produces comparable functional outcomes to the classical Broström-Gould procedure. To our knowledge, there are no prior reports comparing the two approaches with respect to ankle function and stability outcomes.

The purpose of this study was to compare the clinical outcomes in terms of ankle function and stability between repairing or not repairing the ATFL remnant in the arthroscopic modified Broström-Gould procedure. It was hypothesised that the clinical outcomes of ATFL remnant repairing would be similar to those of not repairing at the 2-year follow-up. The results of this study may be clinically useful to identify an optimal option for patients with CLAI in whom the ATFL remnant is not viable when undertaking an arthroscopic modified Broström-Gould procedure.

## Materials and methods

After obtaining institutional review board approval (ID: 2015-078/XZXY-LJ-20161230-021, Xuzhou Central Hospital), data of patients who underwent arthroscopic modified Broström-Gould procedure between 2015 and 2018 were retrospectively reviewed. The investigation was a retrospective cohort study evaluating the clinical outcomes of patients with CLAI undergoing an arthroscopic modified Broström-Gould procedure repairing or not repairing the ATFL remnant. All patients provided a signed informed consent as well as consent under the Health Insurance Portability and Accountability Act to participate in this study pre-operatively and at the completion of 2 years follow-up.

### Patient selection

Inclusion criteria were as follows: (1) CLAI treated with appropriate conservative management for over 6 months, but without improved symptoms; (2) unilateral ankle arthroscopic Broström-Gould procedure with one double-loaded suture anchor fixation (Fastin RC 3.5 mm, Smith & Nephew, Andover, MA); (3) no previous ankle ligament surgery, with no other ankle injury than the index one; (4) complete surgical and follow-up data, and follow-up for at least 24 months.

Exclusion criteria were as follows: (1) combined foot and ankle deformity, abnormal hindfoot alignment, ipsilateral fracture(s) of the foot and ankle, ankylosis and other ligament injuries; (2) combined central and peripheral neuromuscular disease or generalized ligament laxity; (3) ankle osteoarthritis or osteochondral injury requiring osteochondral transplantation; (4) severe underlying disease and inability to tolerate surgery.

### Participants

**Fig. 1:**
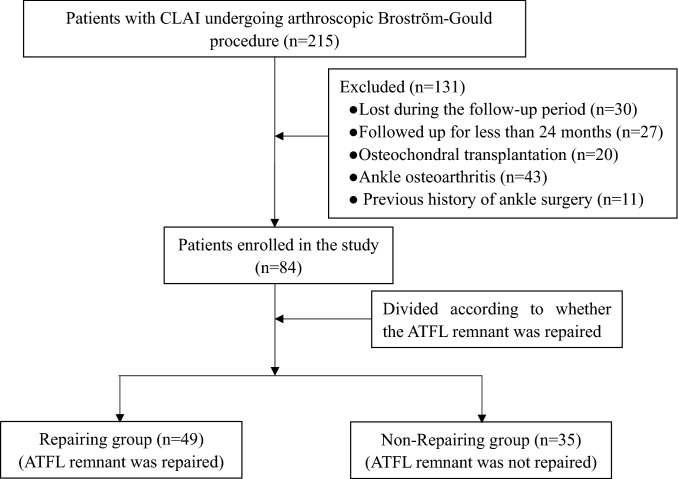
The flow diagram of the study.* CLAI* chronic lateral ankle instability, * ATFL* anterior talofibular ligament

From 2015 to 2018, 215 consecutive patients with CLAI underwent ankle arthroscopic Broström-Gould procedure by a fellowship-trained foot and ankle surgery senior surgeon. Of these, 30 patients were lost to follow-up, and 27 patients were followed up for less than 24 months. Twenty patients with osteochondral injuries were treated with osteochondral transplantation, and 43 patients had ankle osteoarthritis. Eleven patients underwent the Broström-Gould procedure as a revision surgery for CLAI. Eventually, a total of 84 patients with CLAI who underwent arthroscopic Broström-Gould procedure entered the present study (Fig. 1).

According to whether the ATFL remnant had been repaired, the patients were divided into two groups. In the ATFL remnant repair group (*n* = 49), the ATFL remnant and the capsule were repaired and the extensor retinaculum was transferred to strengthen the lateral ankle ligament in the Broström-Gould procedure. In the ATFL remnant non-repair group (*n* = 35), the ATFL remnant was not sutured, either because the quality of the ATFL remnant was poor, it had merged with the ankle joint capsule without indentation, or the preexisting ATFL had been damaged beyond the ability to suture it.

There were no significant differences in the general pre-operative data, which were routinely obtained as part of pre- and post-operative ankle stability assessments, between the two groups in terms of preoperative Visual Analogue Scale (VAS) score, American Orthopedic Foot and Ankle Society (AOFAS) scores, Karlsson Ankle Functional Score (KAFS), or Anterior Talar Translation (ATT) (Table 1).

**Table 1:** Characterization of the sample

Variable	ATFL repair group (*n* = 49)	ATFL non-repair group (*n* = 35)	*P*^a^ value
Age, year	33.3 ± 8.4	35.6 ± 9.9	n.s
Sex			n.s
Male	32	21	
Female	17	14	
BMI, kg/m^2^	25.0 ± 2.5	23.8 ± 3.2	n.s
OCD	18	13	n.s
VAS	6.1 ± 1.8	6.3 ± 1.9	n.s
AOFAS	69.7 ± 9.5	72.7 ± 9.3	n.s
KAFS	66.6 ± 9.2	68.9 ± 10.4	n.s
ATT, mm	7.2 ± 1.6	7.5 ± 1.8	n.s
Disease duration, mo	15.8 ± 3.0	16.3 ± 3.1	n.s

*BMI* Body Mass Index, *OCD* Osteochondral Defect, *VAS* Visual analogue scale, *AOFAS* American Orthopaedic Foot and Ankle Society, *KAFS* Karlsson Ankle Function Score, *ATT* Anterior Talar Translation, *n.s.* non-significant

### Surgical technique

With the patient supine, a 7 cm pillow was placed under the ipsilateral hip after induction of anesthesia. The affected leg was placed over the distal edge of the operating table, and a pneumatic tourniquet placed on the thigh was inflated to 300 mmHg after exsanguination of the limb.

Standard anterolateral and anteromedial ankle arthroscopy portals were established. The intra-articular lesions were fully evaluated and managed. Subsequently, an accessory anterior portal at the fibular tip was established to examine the ATFL, debride the surrounding tissues, and expose and debride the ligament footprint region on the fibula. A double-loaded suture anchor (Fastin RC 3.5 mm, Smith & Nephew, Andover, MA) was inserted into the mid-portion of the footprint area of the fibula. To reduce the number of knots, we sutured the ATFL, lateral capsular and inferior extensor retinaculum together by passing the suture thread directly through them.

**Fig. 2:**
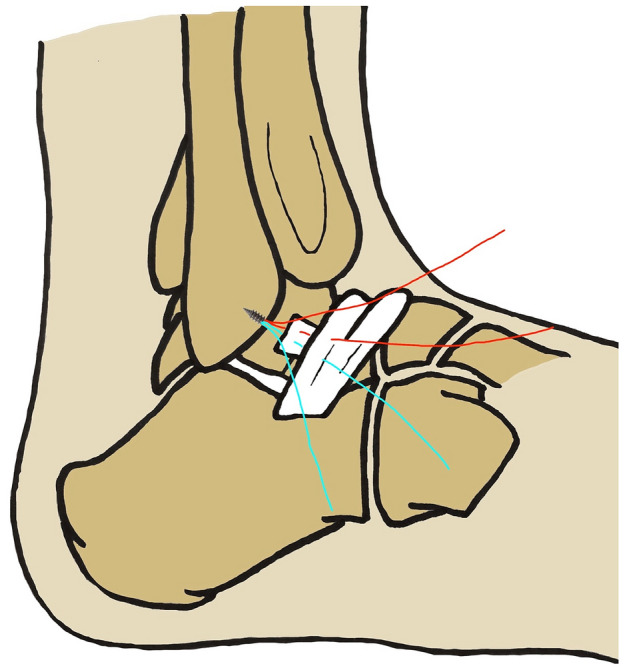
Surgical diagrams of arthroscopic Broström-Gould procedure with anterior talofibular ligament remnant repair

**Fig. 3:**
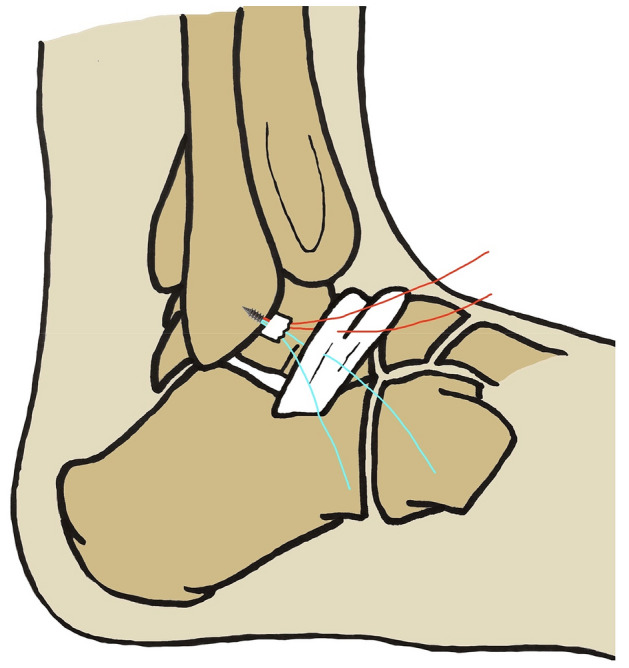
Surgical diagrams of arthroscopic Broström-Gould procedure with anterior talofibular ligament remnant non-repairing

Under arthroscopic visualization, if the ATFL remnant was viable and in continuity, and could be repaired directly, the anchor suture limbs were passed through the ATFL, capsule and inferior extensor retinaculum (Fig. 2). If the ATFL remnant was nonviable or was merged with the ankle joint capsule without indentation, sutures were passed only through the ankle capsule and inferior extensor retinaculum (Fig. 3). With the ankle kept at 5° of eversion, the suture knot was tightened with a knot pusher. The entry portals were sutured in a standard fashion with a non-absorbable monofilament.

### Postoperative management

A splint was used to immobilize the ankle joint for 2 weeks in mild dorsiflexion and eversion. On the second day after surgery, patients were advised to perform early non-weight-bearing functional exercises and isometric exercises of the lower limb. The skin sutures were removed 2 weeks after the procedure, an Aircast™ boot (DJO, Vista, CA, USA) was applied, and weight-bearing functional exercises were started. Physical activities were encouraged after removing the Aircast boot 6 weeks later.

### Postoperative follow-up and observational indexes

Wound healing and ankle stability were assessed after surgery, and the VAS, AOFAS, KAFS, ATT and return to sports were evaluated to assess ankle function. A 10-point VAS was used for assessment, with 0 for no pain, and 10 for the worst possible pain imaginable. All the patients received the guidance of a health care professional before filling in the AOFAS Ankle-Hindfoot Scale score. The KAFS system was used to evaluate the functional recovery after the index procedure. The KAFS consists of eight parts: pain (20 points), swelling (10 points), subjective instability (25 points), stiffness (5 points), stair climbing (10 points), running (10 points), work activities (15 points), and support (5 points) [9]. Ankle proprioception was assessed using the Active Joint Position Sense (AJPS) [1] using the active joint angle reproduction test. The patients were seated on a height-adjustable table with the affected foot placed at a 90° angle from the hip, knee, and ankle. The affected ankle was passively placed in 10° and 20°of inversion and plantar flexion, respectively, three times, using the footplate. Patients were then asked to actively place the foot in these positions. All measurements were made at 1 year and 2 years after surgery, and evaluated by the same experienced rehabilitation physician who did not participate in surgery and was blinded to the surgical procedure performed.

### Statistical analysis

SPSS Version 25.0 software (SPSS, Inc., Chicago, IL, USA) was used to analyze the data. The quantitative variables were expressed as mean ± standard deviation. The measurement data (VAS, AOFAS, KAFS, ATT and AJPS scores) before and after surgery and between two groups after surgery were compared using the Student’s *t*-test (for normal distribution) or the Mann–Whitney test (for asymmetric distribution). The VAS, AOFAS, KAFS, ATT data before and after surgery in each group were compared using the Friedman test or Repeated Measurement ANOVA. The Pearson Chi-square test was used to compare categorical variables. Given the standard deviation of the functional scores (AOFAS or KAFS) in the data set, the sample sizes of the ATFL remnant repair group (*n* = 49) and the ATFL remnant non-repair group (*n* = 35) yielded a power of 85% when the level of significance was set at 0.05. The post hoc power analysis showed that group sample sizes of 49 and 35 achieve less than 20.0% power (VAS, AOFAS, KAFS, ATT and AJPS, respectively) to reject the null hypothesis of equal means, with a significance level (alpha) of 0.05 using a two-sided two-sample unequal-variance *t*-test. Differences of *p* < 0.05 were considered statistically significant.

## Results

Debridement of the synovial tissue of the ankle joint was performed in all patients, and microfracture of the talus was performed in 31 patients (18 in the repair group vs 13 in the non-repair group). These patients in each group were compared with the rest of the patients, and no difference was identified in the evaluation items before and after surgery (Table 2). Osteochondral injury patients in the non-repair group treated with microfracture achieved better AJPS (Inversion 10°) at 1 year compared to those without osteochondral injury. AJPS (Inversion 20°) at 1 year and AJPS at 2 years were not significantly different regardless of whether microfracture surgery had been performed (Table 2). All surgical incisions healed uneventfully. There were no wound complications, reactions to the implants, or neurological or vascular injuries. The operative time and length of hospital stay between the two groups were comparable. Significant improvements in all the functional scores [VAS (repair group, *p* < 0.001; non-repair group, *p* < 0.001), AOFAS (repair group, *p* < 0.001; non-repair group, *p* < 0.001), KAFS (repair group, *p* < 0.001; non-repair group, *p* < 0.001) and ATT (repair group, *p* < 0.001; non-repair group, *p* < 0.001)] were recorded at 1 year and 2 years after surgery. In both groups, results were comparable for VAS, AOFAS, KAFS, ATT and AJPS at 1 year and 2 years (Table 3).

**Table 2:** Functional outcomes comparison of the microfracture and non-microfracture patients of the two groups

	Variable			Microfracture group	Non-microfracture group	*P*^a^ value
Repair group (*n* = 49)				(*n* = 18)	(*n* = 31)	
Pre-	VAS			6.4 ± 2.2	5.9 ± 1.5	n.s
	AOFAS			71.2 ± 11.0	68.8 ± 8.5	n.s
	KAFS			65.2 ± 7.5	67.4 ± 10.1	n.s
Post-	VAS	1 year		0.0 (0.0 ~ 2.0)	0.0 (0.0 ~ 2.0)	n.s
		2 year		0.0 (0.0 ~ 1.0)	0.0 (0.0 ~ 1.0)	n.s
	KAFS	1 year		88.6 ± 4.1	90.7 ± 4.0	n.s
		2 year		92.2 ± 3.7	90.5 ± 4.5	n.s
	AOFAS	1 year		90.3 ± 4.7	90.9 ± 4.7	n.s
		2 year		90.9 ± 4.9	92.3 ± 4.3	n.s
	ATT	1 year		2.5 ± 1.2	2.6 ± 1.2	n.s
		2 year		2.8 ± 1.3	2.9 ± 1.2	n.s
	AJPS	1 year	Inversion 10°	7.4 ± 1.3	7.7 ± 1.3	n.s
		1 year	Inversion 20°	17.6 ± 1.5	17.9 ± 1.7	n.s
		1 year	Plantar flexion 10°	7.6 ± 1.0	7.7 ± 1.3	n.s
		1 year	Plantar flexion 20°	18.6 ± 1.1	18.4 ± 1.4	n.s
		2 year	Inversion 10°	8.4 ± 1.5	7.9 ± 1.6	n.s
		2 year	Inversion 20°	18.1 ± 1.6	17.9 ± 1.7	n.s
		2 year	Plantar flexion 10°	8.0 ± 1.1	7.7 ± 1.3	n.s
		2 year	Plantar flexion 20°	18.9 ± 1.3	18.5 ± 1.3	n.s
Non-repair group (*n* = 35)				(*n* = 13)	(*n* = 22)	
Pre-	VAS			6.2 ± 1.3	6.4 ± 2.3	n.s
	AOFAS			70.7 ± 8.3	73.9 ± 9.8	n.s
	KAFS			64.5 ± 6.6	71.6 ± 11.4	n.s
Post-	VAS	1 year		0.0 (0.0 ~ 2.0)	0.0 (0.0 ~ 2.0)	n.s
		2 year		0.0 (0.0 ~ 1.0)	0.0 (0.0 ~ 1.0)	n.s
	KAFS	1 year		90.4 ± 4.5	90.0 ± 4.7	n.s
		2 year		90.9 ± 4.2	90.1 ± 4.3	n.s
	AOFAS	1 year		91.7 ± 4.5	91.5 ± 4.7	n.s
		2 year		91.4 ± 4.0	92.8 ± 4.8	n.s
	ATT	1 year		2.3 ± 0.8	2.5 ± 0.9	n.s
		2 year		2.5 ± 1.0	2.7 ± 1.0	n.s
	AJPS	1 year	Inversion 10°	8.4 ± 1.5	7.4 ± 1.1	0.02
		1 year	Inversion 20°	17.8 ± 1.6	18.1 ± 1.5	n.s
		1 year	Plantar flexion 10°	7.7 ± 1.3	7.9 ± 1.1	n.s
		1 year	Plantar flexion 20°	18.2 ± 1.4	18.3 ± 1.4	n.s
		2 year	Inversion 10°	7.5 ± 1.1	8.1 ± 1.2	n.s
		2 year	Inversion 20°	18.0 ± 1.6	18.2 ± 1.9	n.s
		2 year	Plantar flexion 10°	7.8 ± 1.4	8.0 ± 1.4	n.s
		2 year	Plantar flexion 20°	17.9 ± 1.6	18.7 ± 1.0	n.s

*VAS* Visual analogue scale, *AOFAS* American Orthopaedic Foot and Ankle Society, *KAFS* Karlsson Ankle Function Score, *ATT* Anterior Talar Translation, *AJPS* Active Joint Position Sense, *n.s.* non-significant

**Table 3:** Functional outcomes comparison of the two groups

Variable	Repair group (*n* = 49)	Non-repair group (*n* = 35)	*p* value	Power^a^
Operative time, min	46.0 ± 7.1	44.0 ± 9.5	n.s	
LHS, day	3.6 ± 0.6	3.4 ± 0.6	n.s	
VAS				
1 year	0.0 (0.0–1.0)	0.0 (0.0–2.0)	n.s	0.06
2 year	0.0 (0.0–1.0)	0.0 (0.0–1.0)	n.s	0.05
AOFAS				
1 year	90.7 ± 4.7	91.5 ± 4.6	n.s	0.1
2 year	91.8 ± 4.5	92.3 ± 4.5	n.s	0.07
KAFS				
1 year	89.9 ± 4.2	90.1 ± 4.5	n.s	0.05
2 year	91.1 ± 4.2	90.4 ± 4.2	n.s	0.1
ATT, mm				
1 year	2.6 ± 1.2	2.4 ± 0.9	n.s	0.08
2 year	2.8 ± 1.2	2.6 ± 1.0	n.s	0.2
AJPS, degree				
1 year				
Inversion 10°	7.6 ± 1.3	7.7 ± 1.3	n.s	0.09
Inversion 20°	17.8 ± 1.6	18.0 ± 1.6	n.s	0.08
Plantar flexion 10°	7.6 ± 1.2	7.8 ± 1.2	n.s	0.1
Plantar flexion 20°	18.5 ± 1.3	18.2 ± 1.4	n.s	0.1
2 year				
Inversion 10°	8.1 ± 1.6	7.9 ± 1.2	n.s	0.1
Inversion 20°	18.0 ± 1.7	18.1 ± 1.7	n.s	0.08
Plantar flexion 10°	7.8 ± 1.2	7.9 ± 1.4	n.s	0.07
Plantar flexion 20°	18.7 ± 1.3	18.4 ± 1.3	n.s	0.1

*LHS* Length of hospital stay, *VAS* Visual analogue scale, *AOFAS* American Orthopaedic Foot and Ankle Society, *KAFS* Karlsson Ankle Function Score, *ATT* Anterior Talar Translation, *AJPS* Active Joint Position Sense, *n.s.* non-significant

^a^Power is computed to reject the null hypothesis of equal means

The mean time to the return to normal activity for patients in the repair group was 8.2 ± 2.4 (range 6–10) weeks versus 8.4 ± 3.1 (range 6–10) weeks for those in the non-repair group (not significant). At final follow-up, in the repair group, 34 patients had resumed the pre-injury sports activities and 15 patients were involved in leisure sports activities only because of fear of secondary injury to the surgery site; in the non-repair group, 24 people returned to pre-injury sports activities and 11 people were involved in leisure sports activities only because of fear of secondary injury to the surgery site.

## Discussion

The main finding of the current study was that repairing or not repairing the ATFL remnant gave comparable functional outcomes following the arthroscopic Broström-Gould procedure for CLAI. There were no differences in VAS, AOFAS, KAFS, ATT, AJPS, time of return to normal activity, or the rate of return to pre-injury sports between procedures.

The arthroscopic Broström-Gould procedure is widely used. It involves repair of the lateral ligament complex, and uses the extensor retinaculum to strengthen the repair; at arthroscopy, the intra-articular lesions are addressed, with results comparable to the open Broström-Gould procedure [3, 16, 23]. The classical Broström-Gould procedure has two steps: first, suture of the ATFL and joint capsule repair; second, the imbrication of the extensor retinaculum and suture it over the anterior edge of the fibula [2, 8]. Xu et al. [24] retrospectively compared the outcomes of 53 patients with CLAI treated with the arthroscopic assisted Broström-Gould procedure (28 patients) or with augmentation using suture tape (25 patients). The capsule, ATFL remnant, and extensor retinaculum were securely fixed with anchors. After a follow-up of 2 years, both groups achieved adequate stability, and the patients returned to exercise activity without difficulties. Lee et al. [12] performed arthroscopic and open Broström-Gould surgery on 11 matched pairs of human cadaver specimens. The operative procedure involved ATFL imbrications with inferior extensor retinaculum reinforcement. The arthroscopic Broström-Gould surgery was a reasonable alternative procedure for CLAI, with similar biomechanical stability to classic Broström-Gould surgery.

At times, the ATFL remnant can be merged with the ankle joint capsule, or the ATFL may have been damaged beyond suture, and the capsule and extensor retinaculum are the only viable tissues [10]. In these instances, the ATFL cannot be repaired, poor ligament tissue quality can limit repair techniques, and reconstruction with allograft or autograft would be considered [21]. Song et al. [19] reported 12 consecutive patients with CLAI treated using an all-inside arthroscopic anatomical ATFL reconstruction with a semitendinosus autografts because the lateral ligament remnants were of a suboptimal quality to allow repair. After 30 months follow-up, the functional results were comparable to those of the modified Broström procedure group.

Lateral ankle ligament reconstruction procedures are a reliable option with acceptable functional outcomes when the torn lateral ankle ligament remnants do not allow a repair [14]. ATFL reconstruction allows restoration of the continuity of the lateral ankle ligament complex and mechanical stability of the ankle. However, ligament reconstruction procedures are more complicated than ligament repair procedures and may require more advanced technical skills. Ligament reconstructions requires longer post-operative proprioceptive functional rehabilitation. Therefore, anatomic repair techniques may offer advantages over reconstruction procedures [4].

Partial defects of the ATFL with subfibular ossicle excision afforded clinical and radiographical outcomes comparable to those in the traditional modified Broström procedure. Kim et al. [11] compared the results of 26 CLAI ankles with subfibular ossicles to those of 99 ankles without subfibular ossicles. The ossicle to the lateral ligament was resected, the ATFL remnants were not repairable, and augmentation of the lateral capsule and inferior extensor retinaculum was performed using the all-inside arthroscopic modified Broström procedure. In the present study, when the ATFL remnants were not repairable, we elected to undertake a modified Broström-Gould procedure without suturing the ATFL. Patients in whom the ATFL remnant had not been repaired can achieve similar ankle functional stability to those who undergo ATFL remnant repair, with comparable post-operative proprioceptive function between the two groups. These findings suggest that, if the ATFL remnant is not viable, a modified Broström-Gould procedure without ATFL suturing can still be performed instead of a more complex lateral ligament reconstruction.

All the patients were followed up for an average of 2 years. This may be considered a relatively short time. Nevertheless, by this point, the results of surgery would have stabilized, and recovery would be effected. Furthermore, this length of follow-up allowed us to minimize the number of patients defaulting from the study: it would have been difficult to ask patients to return for assessment several years later after the index procedure.

When considering the suture fashion, Maffulli et al. [15] using a vest-over-pant suture fashion to plicate the ATFL, and Nery et al. [16] repaired the ATFL and inferior extensor retinaculum in layers. We sutured the ATFL, lateral capsular and inferior extensor retinaculum together by passing the suture directly through them. All the above suture configurations produced satisfactory ankle functional results: one suture configuration cannot be recommended over any other. Surgeons should use whichever configuration they feel comfortable with.

This study has several strengths: (1) it carefully assessed post-operative ankle function, stability and proprioception; (2) it recruited a representative sample of patients; (3) all the procedure were performed by a single highly qualified surgeon; and (4) patients underwent a standardized rehabilitation programme. Nevertheless, several limitations should also be noted: the measurement method for the proprioceptive function was simple, and might not comprehensively assess all aspects of ankle proprioception. The osteochondral injuries requiring microfracture might potentially confound the interpretation of the main study question regarding ATFL repair versus no repair, although the results of this study showed no difference. Another limitation was that, as a retrospective cohort study, there was potential patient selection bias. This limitation was compensated, to some extent, by the fact that all the procedures were performed by the same senior surgeon, all outcomes were evaluated by an experienced ankle surgeon who was not involved in the selection of patients and their surgical care, and the postoperative functional exercise was performed under a guidance of a rehabilitation specialist.

To our knowledge, this is the first study which showed comparable clinical outcomes between repairing and not repairing the ATFL remnant following the arthroscopic Broström-Gould procedure for CLAI: whether the ATFL remnant is repair or not, the all-inside arthroscopic Broström-Gould procedure for CLAI yields equally good results.

## Conclusion

Surgeons who have extensive experience with arthroscopic Broström ankle procedures for CLAI may obtain similar ankle stability and function results up to 2 years regardless of whether the procedure includes repair of the ATFL.

## References

[1] Bae YS (2017). Effects of spiral taping on proprioception in subjects with unilateral functional ankle instability. J Phys Ther Sci.

[2] Buerer Y, Winkler M, Burn A, Chopra S, Crevoisier X (2013). Evaluation of a modified Broström-Gould procedure for treatment of chronic lateral ankle instability: a retrospective study with critical analysis of outcome scoring. Foot Ankle Surg.

[3] Cao Y, Hong Y, Xu Y, Zhu Y, Xu X (2018). Surgical management of chronic lateral ankle instability: a meta-analysis. J Orthop Surg Res.

[4] Dierckman BD, Ferkel RD (2015). Anatomic reconstruction with a semitendinosus allograft for chronic lateral ankle instability. Am J Sports Med.

[5] Ferran NA, Maffulli N (2006). Epidemiology of sprains of the lateral ankle ligament complex. Foot Ankle Clin.

[6] Ferran NA, Oliva F, Maffulli N (2009). Ankle instability. Sports Med Arthrosc Rev.

[7] Golanó P, Vega J, de Leeuw PA, Malagelada F, Manzanares MC, Götzens V, van Dijk CN (2010). Anatomy of the ankle ligaments: a pictorial essay. Knee Surg Sports Traumatol Arthrosc.

[8] Gould N, Seligson D, Gassman J (1980). Early and late repair of lateral ligament of the ankle. Foot Ankle.

[9] Karlsson J, Peterson L (1991). Evaluation of ankle joint function: the use of a scoring scale. The Foot.

[10] Keller M, Grossman J, Caron M, Mendicino RW (1996). Lateral ankle instability and the Brostrom-Gould procedure. J Foot Ankle Surg.

[11] Kim WJ, Lee HS, Moon SI, Kim HS, Yeo ED, Kim YH, Seok Park E, Lee YK (2019). Presence of subfibular ossicle does not affect the outcome of arthroscopic modified broström procedure for chronic lateral ankle instability. Arthroscopy.

[12] Lee KT, Kim ES, Kim YH, Ryu JS, Rhyu IJ, Lee YK (2016). All-inside arthroscopic modified Broström operation for chronic ankle instability: a biomechanical study. Knee Surg Sports Traumatol Arthrosc.

[13] Lee KT, Park YU, Kim JS, Kim JB, Kim KC, Kang SK (2011). Long-term results after modified Brostrom procedure without calcaneofibular ligament reconstruction. Foot Ankle Int.

[14] Li Q, Ma K, Tao H, Hua Y, Chen S, Chen S, Zhao Y (2018). Clinical and magnetic resonance imaging assessment of anatomical lateral ankle ligament reconstruction: comparison of tendon allograft and autograft. Int Orthop.

[15] Maffulli N, Del Buono A, Maffulli GD, Oliva F, Testa V, Capasso G, Denaro V (2013). Isolated anterior talofibular ligament Broström repair for chronic lateral ankle instability: 9-year follow-up. Am J Sports Med.

[16] Nery C, Raduan F, Del Buono A, Asaumi ID, Cohen M, Maffulli N (2011). Arthroscopic-assisted Broström-Gould for chronic ankle instability: a long-term follow-up. Am J Sports Med.

[17] Řezaninová J, Hrazdira L, Moc Králová D, Svoboda Z, Benaroya A (2018). Advanced conservative treatment of complete acute rupture of the lateral ankle ligaments: verifying by stabilometry. Foot Ankle Surg.

[18] Rigby RB, Cottom JM (2019). A comparison of the "All-Inside" arthroscopic Broström procedure with the traditional open modified Broström-Gould technique: a review of 62 patients. Foot Ankle Surg.

[19] Song B, Li C, Chen N, Chen Z, Zhang Y, Zhou Y, Li W (2017). All-arthroscopic anatomical reconstruction of anterior talofibular ligament using semitendinosus autografts. Int Orthop.

[20] Song Y, Li H, Sun C, Zhang J, Gui J, Guo Q, Song W, Duan X, Wang X, Wang X, Shi Z, Hua Y, Tang K, Chen S, Chinese Society of Sports Medicine (2019). Clinical guidelines for the surgical management of chronic lateral ankle instability: a consensus reached by systematic review of the available data. Orthop J Sports Med.

[21] Teixeira J, Guillo S (2018). Arthroscopic treatment of ankle instability - allograft/autograft reconstruction. Foot Ankle Clin.

[22] Vasta S, Papalia R, Albo E, Maffulli N, Denaro V (2018). Top orthopedic sports medicine procedures. J Orthop Surg Res.

[23] Xu C, Li M, Wang C, Liu H (2020). A comparison between arthroscopic and open surgery for treatment outcomes of chronic lateral ankle instability accompanied by osteochondral lesions of the talus. J Orthop Surg Res.

[24] Xu DL, Gan KF, Li HJ, Zhou SY, Lou ZQ, Wang Y, Li GQ, Ruan CY, Hu XD, Chen YL, Ma WH (2019). Modified Broström repair with and without augmentation using suture tape for chronic lateral ankle instability. Orthop Surg.

[25] Zeng G, Hu X, Liu W, Qiu X, Yang T, Li C, Song W (2020). Open Broström-Gould repair vs arthroscopic anatomical repair of the anterior talofibular ligament for chronic lateral ankle instability. Foot Ankle Int.

